# Supplementary material for: Modelling of magnetic vortex microdisc dynamics under varying magnetic field in biological viscoelastic environments
Source: Nanoscale Adv. 2026 Jan 5;8(5):1570–84. doi: 10.1039/d5na00854a (PMC12848370; doi:10.1039/d5na00854a)
Supplement: NA-008-D5NA00854A-s001 [file NA-008-D5NA00854A-s001.pdf]

Supplementary Information (SI) for Nanoscale Advances.
This journal is © The Royal Society of Chemistry 2026

Supplementary information

Modelling of magnetic vortex microdisc dynamics under varying magnetic field in biological viscoelastic environments

Andrea Visonà[1,2], Robert Morel[2], Hélène Joisten[2], Bernard Dieny[2], and Alice Nicolas[1]

[1]Univ. Grenoble Alpes, CNRS, CEA/LETI-Minatec, Grenoble INP, LTM, Grenoble F-38000, France.
[2]Univ. Grenoble Alpes, CEA, CNRS, Spintec, Grenoble F-38000, France.

1 Derivation of the demagnetising energy term

Writing the second Maxwell equation in the absence ocellular alterations following stimulation with such particles are to be attributed to mechanical origin external field yields $\nabla \cdot B = \mu_0 \nabla \cdot (H_d + M) = 0$ and hence $\nabla H_d = -\nabla \cdot M$, with B the magnetic induction, H_d the demagnetising field, M the magnetisation and μ_0 the free space permeability. Note that here, only the part of the dipolar field occurring inside the magnetic body, the so-called demagnetising field, is considered. From this equation, it is possible to define magnetic volume charges as $\rho_m = -\nabla \cdot M$ in the bulk and surface charges $\sigma_m = \mathbf{M} \cdot \hat{n}$ where $\hat{n}$ is the normal to the surface. When considering only the dipolar interaction, the ground state is then given by the magnetic configuration that minimises the volume and magnetic surface charges, that will minimise the demagnetising field and therefore minimises the energy. Thus, the shape of the sample strongly influences the demagnetising energy, giving rise to an anisotropy referred to as shape anisotropy and some preferential axis or planes where the magnetisation prefers to lay. To illustrate this, let us consider the case of a uniformly magnetised thin ellipsoid with principal axis along (x, y, z) such that the semiaxis R >> h.

The demagnetising field can be expressed simply in terms of the demagnetising coefficients N_i as:

$$H_{d,i} = -N_i M_i \tag{S1}$$

In the chosen geometry the tensor N has non-zero terms only in the diagonal and its trace is equal to 1. The demagnetising field in polar spherical coordinates (θ, ϕ), reads as:

$$H_d = -N\mathbf{M} = -\begin{pmatrix} N_R & 0 & 0 \\ 0 & N_R & 0 \\ 0 & 0 & N_h \end{pmatrix} M_{sat} \begin{pmatrix} \cos\phi \sin\theta \\ \sin\phi \sin\theta \\ \cos\theta \end{pmatrix} = -M_{sat} \begin{pmatrix} N_R \cos\phi \sin\theta \\ N_R \sin\phi \sin\theta \\ N_h \cos\theta \end{pmatrix} \tag{S2}$$

The energy is calculated as:

$$\varepsilon_d = -\frac{1}{2}\mu_0 \mathbf{M} \cdot \mathbf{H}_d = \frac{1}{2}\mu_0 M_{sat}^2 \begin{pmatrix} \cos\phi \sin\theta \\ \sin\phi \sin\theta \\ \cos\theta \end{pmatrix} \begin{pmatrix} N_R \cos\phi \sin\theta \\ N_R \sin\phi \sin\theta \\ N_h \cos\theta \end{pmatrix} \tag{S3}$$

which becomes:

$$\varepsilon_d = \frac{1}{2}\mu_0 M_{sat}^2 \left(N_R \sin^2\theta + N_h \cos^2\theta\right) \tag{S4}$$

with the relation $N_R = \frac{1-N_h}{2}$

We now introduce a more meaningful angle, δ, which is the angle between the easy plane of the ellipsoid and the magnetisation vector and rewrite the demagnetisation energy as function of it. Indeed δ allows to easily indicate the in plane and out of plane component of the magnetisation. Being $\delta = \pi/2 - \theta$

$$\varepsilon_d = \frac{1}{2}\mu_0 M_{sat}^2 \left(N_R \cos^2\delta + N_h \sin^2\delta\right) \tag{S5}$$

Expressing the previous equation as energy (E) instead of energy density (ε) we obtain:

$$E_d = \frac{1}{2}\mu_0 \frac{m_{sat}^2}{V} \left(N_R \cos^2\delta + N_h \sin^2\delta\right) \tag{S6}$$

2 Supplementary movies

- Motion of the microdisc in a viscous environment: same as Fig. 8 ($n = 0.2$, $g = 0$), **Supplementary_movie_1.mp4**
- Motion of a microdisc trapped in a predominantly elastic material: same as Fig. 9 ($n = 4 \times 10^{-3}$, $g = 2$), **Supplementary_movie_2.mp4**
- Motion of a microdisc in a viscoelastic material, the viscous friction being comparable to the elastic resistance: same as Fig. 10 ($n = 0.1$, $g = 0.2$), **Supplementary_movie_3.mp4**
